# Scale-up of Global Child and Youth Mental Health Services: A Scoping Review

**DOI:** 10.1007/s10488-024-01400-3

**Published:** 2024-08-06

**Authors:** Sarah Cusworth Walker, Lawrence Wissow, Noah R. Gubner, Sally Ngo, Peter Szatmari, Chiara Servili

**Affiliations:** 1 University of Washington, 4333 Brooklyn Ave NE, Box 359457, Seattle, WA 98195-9457 USA; 2 University of Washington, 4800 Sand Point Way NE, MS OA.5.154, Seattle, WA 98105 USA; 3 University of Toronto, 80 Workman Way, Toronto, ON M6J 1H4 Canada; 4 World Health Organization, Avenue Appia 20, 1201 Geneva, Switzerland

**Keywords:** CYMHS, Child and youth, Scale-up, Scaling, Mental health, Implementation, Mental health

## Abstract

Numerous influential policy and scientific bodies are calling for more rapid advances in the scale-up of child and youth mental health services (CYMHS). A number of CYMHS innovations hold promise for advancing scale-up but little is known about how real-world efforts are progressing. We conducted a scoping review to identify promising approaches to CYMHS scale-up across the globe. Searches were completed in six databases (Academic Search Complete, CINAHL, MEDLINE, PsychInfo, PubMed, and Web of Science). Article selection and synthesis were conducted in accordance to the Preferred Reporting Items for Systematic Reviews and Meta-Analysis extension for Scoping Reviews (PRISMA-ScR) checklist. A second search focused on low-and-middle-income countries (LMIC) was conducted based on the Cochrane Library recommended search filters of the World Bank listed LMIC countries. Authors used a double coding strategy during the title/abstract and full-text review. Twenty-eight articles meeting the eligibility criteria were identified that described 22 initiatives (in 11 different countries). Our review found the majority of published scale-up studies in CYMHS were not informed by scale-up frameworks in design or reporting. The methods and outcomes used in the identified articles were highly variable and limited our ability to draw conclusions about comparative effectiveness although promising approaches emerged. Successes and failures identified in our review largely reflect consensus in the broader literature regarding the need for strategies to better navigate the complexities of system and policy implementation while ensuring CYMHS interventions fit local contexts.

## Introduction

Unmet need for mental health care among children and youth is a global health crisis. The worldwide prevalence of any mental disorder among children and youth is roughly 10% (Erskine et al., 2017; Kieling et al., 2024; Polanczyk et al., 2015). Analyses from the Global Burden of Disease consortium confirm that depression and anxiety account for the top disorders associated with disability among adolescents (Whiteford et al., 2013). High quality child, adolescent, and young adult treatment can prevent long-term chronic mental health and physical health disabilities (Pilling et al., 2020), but children and youth are dramatically underserved in countries across the income spectrum (Kieling et al., 2011). UNICEF estimates that only 2% of national budgets are devoted to children and youth mental health services, worldwide (United Nations Children's Fund (UNICEF), 2021).

### Scale Up Frameworks in Healthcare

The scale up of evidence-based mental health services is a global health priority among policy, research, and funding organizations including the World Health Organization (WHO), the US National Institute of Mental Health, the Canadian Department for International Development (DFID), and the Wellcome Trust. To date, published reviews and studies of adult mental health scale-up efforts identify common barriers, including cultural differences in the view of mental health disorder, health worker motivation, staff turnover, lack of finances, and lack (or presence) of institutional and policy support (Keynejad et al., 2021). Recommendations from the WHO Mental Health Gap Action Programme (mhGAP) note the need for scale-up efforts to be more richly informed by reflective questions about local conceptions of wellness, systems of care, and values (Gómez-Carrillo et al., 2020). Approaches to these barriers within mental health services scale up reflect many of the recommendations in scale-up frameworks across health areas.

Scale-up in healthcare is generally understood to involve the activities needed to ensure equitable access to high quality services for a specific treatment area and/or client population. Previous studies suggest this requires building or enhancing a service infrastructure (establishing services) and then using that infrastructure to deliver high quality care (diffusing or spreading innovations within a service infrastructure) (Lanham et al., 2013). For simplicity, in this paper we use the term “scale-up” to describe both aims. Slight variations in scale-up models reflect the respective concerns of different philosophies within health services research including implementation science (Barker et al., 2016), complexity and realist evaluation theory (Lanham et al., 2013; Willis et al., 2016), and social justice (Sánchez Rodríguez et al., 2021).

### High Quality Children and Youth Mental Health Services (CYMHS)

While child and youth mental health are included in global health priorities, little is known about whether and how the approach to scale up may be different for CYMHS treatment than scale up for adult populations. High quality CYMHS approaches, as articulated by a number of global and national expert organizations (the World Health Organization, the World Economic Forum (with Orygen), the UK Quality Care Commission, and the U.S. National Academies of Science, Engineering, and Medicine), increasingly emphasize the importance of family systems approaches in addressing a variety of child and youth mental health needs. A family systems approach, in contrast to individual treatment, requires shifts in traditional funding (Howe et al., 2014; Hughes et al., 2018; McGorry et al., 2013; NASEM, 2017; Ordóñez & Collins, 2015), and engaging multiple child-serving systems in the provision of quality care. Consequently, it would be a mistake to presume that efforts to scale up mental health services for adult populations would automatically improve child and youth services as well. Little is known about the activities and capacities needed to bring quality child and youth mental services to scale across multiple sites and systems.

### Current Study

To assess the current state of research on efforts to scale up CYMHS, we conducted a scoping review of studies from high-, low- and middle-income countries. The purpose of the review was to identify promising methods and models for scaling up CYMHS to inform implementation and research efforts in this area. The review was guided by three primary questions: (1) What frameworks or models are informing real world scale-up efforts in child and youth mental health?; (2) What outcomes are being measured and what are the preliminary findings of these efforts?; (3) What specific implementation strategies are being used to advance scale-up? Coding for the third question was informed by Sánchez-Rodríguez’s scalability framework (Sánchez-Rodríguez et al., 2021), (Table 1) for its comprehensiveness regarding existing scalability frameworks and its focus on equity-informed implementation. We also drew from the Expert Recommendations for Implementing Change taxonomy (Powell et al., 2015). We adopted a scaling framework in order to align results from our coding with pre-existing knowledge in this field. The Sánchez-Rodríguez et al. (2021) framework is informed by a conceptual review of 20 scale-up frameworks. From their synthesis of existing frameworks, the authors proposed four phases of scaling with four dimensions of planning occurring within each phase. This includes efforts involved scaling “up” (intentional efforts to influence policy); scaling “out” (resources devoted to communicating and supporting buy in); scaling “in” (site capacity building); and scaling “down” (efforts to devolve power to local communities).

**Table 1:** Health scalability frameworks

Author	Year	Health area	Theoretical influence	Core components
Barker, Reid & Schall	2016	General health. Case studies in the prevention of mother-to-child HIV transmission, and maternal child health programs	Systematic review of scale-up in health care. Models and frameworks from Associates in Process Improvement (API) and the Institute for Healthcare improvement (IHI). Two national scale improvement initiatives in Ghana and South Africa	(1) Set-up; (2) Develop the Scalable Unit; (3) Test of Scale-up; (4) Go to Full Scale
Lanham, Leykum, Taylor, Cannon, Lindberg & Lester	2013	General healthcare. Case studies in HIV ART adherence in HIV-infected clients and MRSA prevention in hospitals	Complexity science. Two case studies with multiple sites, one in Kenya and one in the United States	(1) Self-organization vs. imposed structure; (2) Interdependencies; (3) Sensemaking
Willis, Riley, Stockton, Abramowicz, Zummach, Wong, Robinson & Best	2016	General healthcare. Case studies in community mobilization, anti-poverty programs	Realist synthesis informed by three scale up efforts: Vibrant Communities (Canadian national program), Pathways to Education (17 communities), YouthBuild (260 programs)	Four mechanisms: (1) Awareness, (2) Confidence, (3) Commitment, 4) Trust. Activities that activate mechanisms: Renew and regenerate, Demonstrate success
Sánchez Rodriguez, MacLachlan & Brus	2020	Social innovation	Social inclusivity to ensure marginalized population are not 'left behind' in scale up efforts. Review of 20 scale up frameworks	Four directions: (1) Up (change in laws, policies, norms); (2) Down (resource allocation for implementation); (3) In (support organizational capacity); (4) Out (replication or broadening scope). Pathway to scaling: Identifying, Planning, Implementing, Learning, and Adapting

A breakdown of the implementation strategies used to anticipate barriers to scale-up of child and youth mental health services (CYMHS). This includes the health areas, theoretical influences, and the core components

## Methods

The review included published studies focused on efforts scale child and youth (age 5–24 years old) mental health services. The review was primarily interested in strategies to scale at the country or population level but also considered studies that focused on regional areas governed by central policy bodies (state, district). An additional qualifier of the review was a focus on scaling up “quality” mental health services to reflect the aims of the World Health Organization to promote youth and client-friendly, community-based services that are effective in treating mental health needs. Quality services as defined by this review was guided by principles articulated by the WHO in publications (World Health Organization (WHO, 2022) and included studies that were explicit in focusing on one or more of the following principles: Human rights principles, accessibility, effectiveness, person-centeredness, recovery-oriented, and responsiveness to the needs of children, youths, and their families. Evidence from controlled clinical trials was not necessary for an intervention to be defined as a “quality service” but the intentional use of an evidence-supported treatment was a coding element in document coding. The present review was registered by the Open Science Framework. More details are found under https://osf.io/vb9ah.

### Search Strategy

Scoping reviews are a methodologically rigorous approach to describing the scholarly literature on a topic of interest (Arksey & O'Malley, 2007). We used the most recent guidance for conducting high quality scoping reviews, drawing from foundational literature (Arksey & O'Malley, 2007) and updated methods (Levac et al., 2010; Pham et al., 2014; Peters et al., 2020). The review was conducted in accordance with the Joanna Briggs Institute methodology for scoping reviews (Peters et al., 2015). Article selection and synthesis were conducted based on the Preferred Reporting Items for Systematic reviews and Meta-Analyses extension for Scoping Reviews (PRISMA-ScR) checklist. Relevant published research articles were identified by a systematic search of the following databases conducted in January 2021: PubMed, Academic Search Complete, Web of Science, and through EBSCO (MEDLINE, PsychInfo, and CINAHL). The search included text words contained in the titles, abstracts, and keywords of articles. All keywords and index terms were adapted for each database and/or information source. For example, this included search terms for children/youth (e.g. child, adolescent, youth, etc.), mental health (psychological, psychiatric, etc.), and scale-up (scaling up, scalable, etc.). An example of the full search string used in PubMed is listed in the appendix.

***Inclusion Criteria.*** The review included empirical studies (including case studies and qualitative studies) published in the last 20 years (1/1/2000–05/01/2023) that described efforts to scale-up quality mental health services for children and youth (age 5–24 years old) with mental health conditions. To be considered, the article needed to indicate an a priori goal to increase the access, coverage, or quality of children/youth mental health services in a region and be implemented across more than two service/delivery sites. The review was focused on identifying: (1) The frameworks or model informing the scale-up effort; (2) The outcomes measured and any preliminary findings; and (3) Any specific implementation strategies or approaches used to advance scale-up.

***Exclusion Criteria.*** The review excluded articles focused on universal mental health promotion and prevention, interventions focused solely on substance use disorders or neurodevelopmental disorders, and epidemiological studies. The review also excluded protocol papers (as the review focused on learning from efforts that been attempted), studies focused primarily on treatment effectiveness rather than achieving scale, studies not focused on child or youth (age 5–24 years old) mental health services, studies focused on implementation factors not directly related to scalability, and studies published before 1/1/2000.

***Secondary supplemental search.*** As the initial review returned very few studies from low-and-middle income countries (LMIC), a second search was conducted to identify additional relevant literature. This search included terms for mental health and specific mental health disorders (e.g., anxiety, trauma, depression, etc.) in the title and included the Medical Subject Headings (MeSH) terms based on the Cochrane Library recommended search filters of the World Bank listed LMIC countries (Cochrane Effective Practice and Organisation of Care (EPOC), 2020). The remaining search strings identified text words contained in the titles, abstracts, and keywords. The second search was conducted in the same six databases as the initial search (Academic Search Complete, PubMed, Web of Science, CINAHL Complete, PsychInfo, and Medline). An example of the search string used for the second search conducted in PubMed is provided in Appendix B.

### Procedure

**Fig. 1:**
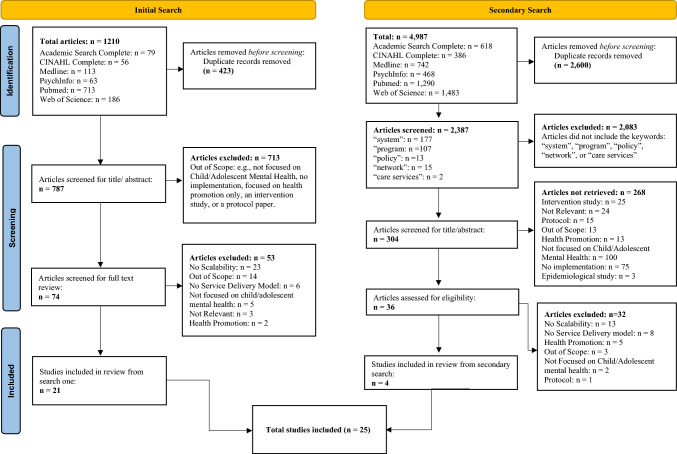
PRISMA

***Data collection and analysis.*** Citations for all identified articles from both searches were collated and uploaded into EPPI-Reviewer (UK 2020) to aggregate and remove duplicates. Article titles were reviewed by two independent reviewers (NG and SN) for inclusion or exclusion based on their titles/abstracts and checked with a third reviewer (SW). The secondary search resulted in many articles that were not relevant to the scoping review, thus, a title keyword search (for the terms “system”, “program”, “policy”, network”, and “care service”) was conducted to pre-screen the articles for the most relevant literature prior to title/abstract review (Fig. 1).

***Number of papers included.*** A total of 1210 articles were identified in the initial search using multiple search databases (Fig. 1). After 423 duplicates were removed, 787 articles were reviewed for inclusion based on title/abstract. A total of 74 articles were found to be eligible for full-text review. After full-text review, 21 articles were found to meet the inclusion criteria and were coded. The secondary search identified 4987 articles, of which 2600 duplicates were removed. Of the remaining 2387 articles, 304 articles contained eligible keywords in the title. After title/abstract review, 36 articles were eligible for full-text coding. After full-text review, 4 articles fit the inclusion criteria and were coded. A total of 28 articles which addressed 22 unique scale-up initiatives from both searches were included and coded for the analyses (Table 2). The most common reason articles were excluded across phases included not being focused on CYMHS, no service delivery model, being focused on universal mental health promotion, being a protocol paper, being an epidemiological or descriptive study, or being a pilot study not yet deployed at scale (Fig. 1).

**Table 2:** Descriptive information and scope of scale-up and client outcome findings for articles

Project	References	Country/region	Scope of scale	Clients served	Client age	Diagnostic target	Scalable unit	Research methods	Outcomes included		References
On Track New York	Mascayano et al. (2019), Nossel et al. (2018)	New York State, USA	21 sites	735–1575	16–30	First episode psychosis	Coordinated specialty care	Pre/post, no comparison	Regional population coverage	Yes	Mascayano (2019)
									Client clinical improvement	Yes	Nossel et al. (2018)
Los Angeles Mental Health County Project	Southam-Gerow (2014), Rodriguez et al. (2018), Reding et al. (2018), Brookman-Frazee (2016)	Los Angeles County, USA	120 sites	27,000	0–25	Multiple mental health needs	Treatment protocol (CBITs, CPP, MAP, TF-CBT, Triple P)	Pre/post, no comparison	Regional population coverage	ND	
									Client clinical improvement	Yes	Brookman-Frazee, (2018), Southam-Gerow (2014)
BASIC—Building and Sustaining Interventions for Children	Dorsey (2019)	Western Kenya	Primary schools in 10 villages	Full BASIC study to train 240 counselors from 40 communities	Children/Adolescents (Not defined)	Trauma (children who experienced parental death)	Group based TFCBT adapted as a task shifting model (delivered by both education and health sectors workers)	Case study	Regional population coverage	ND	
									Client clinical improvement	ND	
Multiple Family Group for Strengthening Families	Choy-Brown (2020)	New York, USA	69 agencies in 5 Burrous of NY City, USA	ND	Children/adolescents (not defined)	Children with behavioral difficulties	The 4 Rs and 2 Ss Multiple Family Group for Strengthening Families	Case study	Regional population coverage	ND	
									Client clinical improvement	ND	
CBITS Connecticut middle schools	Hoover (2018)	Connecticut, USA	State Middle school system	350 kids during a 2-year period	5th-12th grade students	Witnessed or experienced trauma	CBITS	Pre/post, no comparison	Regional population coverage	Yes	Hoover (2018)
									Client clinical improvement	Yes	Hoover (2018)
FRAYME Network	Halsall (2020)	Canada	13 provinces	ND	Services that target youth	Multiple mental health needs	Network designed to accelerate the adoption and scaling up of IYS through evidence & integrated knowledge mobilization	Implementation case study	Regional population coverage	ND	
									Client clinical improvement	ND	
Evidence-based Treatment Dissemination Center (EBTDC)	Hoagwood, (2014)	NY State, USA	State system	Over 2000 providers)	Children/youth (age not defined)	Multiple mental health needs	Multiple system strategies for scaling EBPs	Case study	Regional population coverage	ND	
									Client clinical improvement	ND	
PEPP-Montreal	MacDonald (2018)	Montreal, Canada	City System	1750 referrals over 13 years	14–35	First episode psychosis	Protocol, referrals for First Episode Psychosis (FEP)	Case study	Regional population coverage	Yes, but not clearly described	MacDonald (2018)
									Client clinical improvement	ND	
ACCESS-OM	Malla (2019)	Canada	14 geographically distinct areas	ND	11–25	Multiple mental health needs	MH system transformation model (includes early identification, rapid access, continuity of care)	Case study	Regional population coverage	ND	
									Client clinical improvement	ND	
North Carolina TF-CBT Learning Collaborative	Amaya-Jackson et al. (2018)	North Carolina, USA	Statewide	Project served a large area, but not all clinicians in that area and did not aim to, unclear what proportion of youth were captured	Age not specified. Targeted providers with > 50% clients < 17 years old	Trauma/PTSD, Depression, Externalizing symptoms	TF-CBT	Case study	Regional population coverage	ND	
									Client clinical improvement	Yes	Amaya-Jackson et al. (2018)
Continuing Professional Development programme	Kiima (2010)	Kenya	National	ND	Not defined	Multiple mental health needs	Continuing professional development program to support front line primary care staff to assess, diagnose and manage mental disorders	Implementation case study	Regional population coverage	ND	
									Client clinical improvement	ND	
NY State TA center	Acri (2019)	NY State, USA	Statewide	Served 495 OMH-licensed ambulatory behavioral health clinics	Includes both adult and children specific (or dual) clinics. Though a majority served children	Multiple mental health needs	Technical assistance center for MH professional training and consultation	Implementation case study	Regional population coverage	Yes (but report only on reach for workforce)	Acri (2019)
									Client clinical improvement	ND	
Rolling Cohort—MTFC	Chamberlain (2012)	England	National	ND	Children/adolescents/teenagers	Adolescents referred from juvenile justice or child welfare with severe behavioral problems and are being placed in/or considered for group/residential care	Multidimensional Treatment Foster Care (MTFC)	Implementation case study	Regional population coverage	ND	
									Client clinical improvement	ND	
Cascading model—KEEP	Chamberlain (2012)	San Diego County, USA	County System	ND	Children/adolescents/teenagers	In regular state child welfare foster care at risk or presenting with severe behavioral problems	KEEP	Implementation case study	Regional population coverage	ND	
									Client clinical improvement	ND	
School Mental Health Service (SMHS)	Alonge (2020)	Egypt, Pakistan, Iran	Multi National	As of 2019, trained over 300 public school teachers (6 schools in Irian, 29 in Egypt, and 72 schools in Pakistan)	School aged children (not defined)	Multiple mental health needs	SMHP provided by non specialists	Implementation case study	Regional population coverage	Yes, but limited information	Alonge (2020)
									Client clinical improvement	ND	
Access to Allied Psychological Services (ATAPS)	Bassilios (2014)	Australia	National	Over 10 year period (51,716 clients)	12–25	Multiple mental health needs	ATAPS, a national primary mental health care program	Observational Case study	Regional population coverage	Yes	Bassilios (2014)
									Client clinical improvement	ND	
Skills for Life (SFL)	Guzman (2015)	Chile	National	1007 students referred to specialists (784 engaged in treatment). 1/2 also attended workshops	First graders & third graders (Age 5–8)	1st graders with "psychosocial risk" on screening for acceptance of authority, social contact, cognition, emotional maturity, attention, activity	School-based mental health program	Observational case study	Regional population coverage	Yes	Guzman (2015)
									Client clinical improvement	Yes	Guzman (2015)
SafeCare®	Hurlburt (2014)	CA County, USA (not named)	large County with 3 million residents	ND	Children (age not specified)	Children and families involved with the child welfare system	SafeCare® an evidence-based child neglect prevention program	Cross sectional implementation study	Regional population coverage	ND	
									Client clinical improvement	ND	
Multi County MTFC Implementation (CAL-40 study)	Palinkas et al. (2013), Saldana & Chamberlain (2012)	CA and OH states, USA	51 counties	ND	Youth (not defined)	Youth who have problems with chronic antisocial behavior, emotional disturbance, and delinquency	Multidimensional Treatment Foster Care (MTFC)	Implementation case study	Regional population coverage	ND	
									Client clinical improvement	ND	
Family Check-up (FCU)	Mauricio et al. 2021)	Gothenburg Sweden	14 social services agencies in one large city	ND	10–13	Child problem behaviors	FCU, a brief, assessment-driven intervention	Implementation case study	Regional population coverage	ND	
									Client clinical improvement	ND	
Empowering Parents Empowering Communities (EPEC)	Day (2022)	England	15 sites	Six hundred and eighty-four parents (73.5%) completed the course across the 15 sites	2–5	Parents of 2–5-year-olds with behavioral problems	EPEC is a task sharing, peer-led, group based parenting approach	Implementation case study	Regional population coverage	Yes	Day (2022)
									Client clinical improvement	Significant improvements in positive parenting behavior, equivalent to a large effect size	Day (2022)
Youth FORWARD	Bond (2022)	Sierra Leon, 12 chiefdoms	36 sites	Pilot and scale up programs reached 1783 participants	18–30	Youth facing complex problems, youth exposed to war trauma	Youth FORWARD designed to provide EBP mental health services while advancing goals shared with government and development actors	Implementation case study	Regional population coverage	Yes	Bond (2022)
									Client clinical improvement	No	

Descriptive information for articles included in the review, including scope of scale-up effort and any client outcomes (if reported)

*ND* no data

### Coding Strategy for Scale-Up and Implementation Strategies

The framework used assessed directionality of scaling efforts including scaling “up” (intentional efforts to influence policy); scaling “out” (resources devoted to communicating and supporting buy in); scaling “in” (site capacity building); scaling “down” (efforts to devolve power to local communities). As noted, the coding framework for specific scale-up and implementation strategies was informed by Sanchez-Rodriguez et al.’s scalability model. We also drew from the Standards for Reporting Implementation Studies (STARI, Pinnock et al., 2017) and the Expert Recommendations for Implementing Change (ERIC, Powel et al., 2015) to develop a coding approach for intervention implementation strategies. These two approaches were used to complement each other and covered the range of methodological approaches taken by studies to describe large-scale implementation. Sánchez Rodríguez et al.’s scalability framework ensured information about the methods used to achieve policy change, strategies to achieve reach, and engage local context and ownership (when relevant) was captured, while the STARI and ERIC provided a framework for capturing implementation processes in greater detail within the scaling “in” category of Sánchez Rodríguez’s et al.’s (2021) framework. Risk of bias was not included as it is not the purpose of scoping reviews to draw conclusions about the strength of effects across studies.

Two coders, both PhD level researchers, coded three articles to establish inter-rater reliability. Once the coders had a shared understanding of the coding, the coders randomly selected half of the articles to code. After primary coding was completed, it was reviewed by both coders, and any discrepancies were discussed to achieve consensus. Coding was designed to elicit three levels of information from the published initiatives: (1) Scope of scale-up and client outcome findings (if any) (Table 2); (2) Scale-up approach and scale-up findings (Table 3); Intervention implementation strategies and achieved reach (Table 4). Data was captured in a coding document developed by the reviewers in Microsoft Excel.

**Table 3:** Scale up approach and outcomes

Program	References	Scalable unit	Region	Scale “Up”	Scale “Down”	Scale “In”	Scale “Out”	Scale up findings
On Track New York	Mascayano et al. (2019), Nossel et al. (2018)	Coordinated Specialty Care for First Episode Psychosis	New York State, USA	ND	U.S. federal block grant flow through	Training and TA from a state intermediary (Center for Practice Innovations)	ND	Difficulties with implementation reflect the lack of incentives for coordinating services, service fragmentation, and unclear financial sustainability
LA County Mental Health Project	Southam-Gerow (2014), Rodriguez et al. (2018), Reding et al. (2018), Brookman-Frazee (2016)	Treatment protocols	LA County, USA	Temporary state restriction of mental health funds to innovative mental health services (in context of budget shortfall). A new funding source, the Cal MH Service Act of 2004)	LA County mental health department. LSVFMH (County level) provided implementation support for 6 of the EBPs eligible for reimbursement	Training and TA from treatment protocol developers. Training was ongoing throughout the study time frame; therapists could be trained and begin claiming under PEI at any time	The restriction of service funds to the protocols created motivation for all county-funded mental health sites to participate in training	The quality of scale up was hindered by lack of program and client fit; lack of fit between EBP requirements and client eligibility. For successful sustainment, practice leaders noted the following implementation drivers: the flexibility of the EBP and how it easy it was to use, having an internal champion for the EBP, having an outside entity certify use. Multisite agencies, agencies with more complex client needs, old child clients were associated with greater risk of delivery discontinuation. This highlights the importance of both agency and workforce characteristics in the sustained delivery of EBPs. Potential targets of sustaining interventions include strategic assignment of therapists to EBP training and strategic selection of EBPs by agencies
BASIC—Building and Sustaining Interventions for Children	Dorsey (2019)	Group based TFCBT adapted as a task shifting model (delivered by both education and health sectors workers	Western Kenya	Ministries of Education and health involved in implementation (e.g. providing permission to schools/CHV to participate. Also engaged village leaders so familiar with intervention and could encourage participation	Implementation funded by a NIMH grant. Collaboration between two US universities and Ace Africa	Specific sites were randomly selected. Lay counselors trained and supervised by the experienced lay counselors from the open trial in Tanzania (pilot study), with support from the first author, following the Apprenticeship Model of training. Held 6-day trainings that included didactic instruction, manual review, trainer modeling of group sessions, Q/A, practice w/feedback. Then 2–3 weeks direct support supervision from local trainers	Engaged village leaders so they were familiar with intervention and could encourage participation (details on how this was done were not provided)	Findings from the study suggests a few challenges that would need to be addressed to scale up task shifting for TFCBT. Feasibility of obtaining resources and time for supervision was rated relatively lower in feasibility than other areas (e.g., delivering the content), and there was more variability among teachers in perceived feasibility. But overall, both teachers and health workers felt the program was feasible for their role
Multiple Family Group for Strengthening Families	Choy-Brown (2020)	The 4 Rs and 2 Ss Multiple Family Group for Strengthening Families	New York, USA	Mentions implementation included broad endorsement by state funders and leaders in children's mental health services (though no additional details provided). Their approach seemed more to select a treatment that fits current system. They selected an EBP that is a Medicaid-approved, reimbursable activity and meets the NYS requirements (reimbursable under current structure)	NIH clinical trial that provided no-cost staff training, on-going supervision and consultation, and help facilitating an internal adaptation team	Contact information for all eligible providers provided by the state, study team reached out to invite participation. Agencies identified staff to receive training (4Rs or 2Ss), consisting of 7 online modules followed by a 3 h in-person training (that included behavioral rehearsal of group facilitation). Mentions use of a partnering technical assistance center (but also no description of how this worked). Mentions study included facilitation of an internal adaptation team (though limited details provided on how this worked)	ND	Recommend using an adaptable implementation strategy, that incorporates agency revenue size to scale interventions, and to provide additional resources and support to smaller agencies. Also suggest assisting agencies to appraise the goodness of fit between the setting and the intervention at early stages to avoid drop off. Smaller agencies may be more constrained by limited resources (staffing, money, even physical space) and require financial support combined with training efforts. Such strategies may have potential to facilitate positive EBI adoption decisions and uptake for smaller sized agencies
CBITS Connecticut middle schools	Hoover (2018)	CBITS	Connecticut, USA	Not discussed. Came about as a reaction to the Sandy Hook shooting (so motivated by that tragic event, which likely increased motivation and buy in)	Funding from Connecticut Dept of Children and Families (DCF) and Child Health & Development Institute (CHDI). CHDI is an intermediary organization that supports state to implement and evaluate best practices. Both DCF and CHDI have long history of working together. Strategically targeted getting funding from DCF for this effort	Created two learning communities consisting of teams of 2–6 school-based clinicians trained over 9 months together. Each team received 2-day initial training in CBITS, then participated in bi-weekly clinical consultation calls, received written feedback on 2 audio recorded group sessions (from CBITS trainers), attended 3 follow-up learning sessions for ongoing training and support. Engaged district and school leadership to participate in trainings, and ongoing meetings with DCF, CHDI and CBITS team	Created learning collaboratives. Appeared to engage multiple sites through working with DCF and CHDI (intermediary) and using intentional strategies to engage school leadership to participate in the learning collaborative	Authors note that this study was more successful than other implementation efforts, which they attribute to using a learning collaborative model. Highlight the importance of using strong social networks and collaboration across agencies. Also the importance of strategic targeting of funding, i.e., that the state paid for implementation
Frayme Network	Halsall (2020)	Network designed to accelerate the adoption and scaling up of IYS through evidence & integrated knowledge mobilization	Canada	Frayme network is a strategy to intentionally engage multiple and diverse stakeholders (government officials, philanthropic partners, youth/family members & leaders from MH services)	Funded by Canada's Networks of Centers of Excellence as well as significant support from partners and philanthropic organizations	A participatory needs assessment with diverse stakeholders	NA	The authors conclude that traditional implementation science models are linear and deterministic and not well-suited for systems level change. The authors recommend using frameworks focused on systems and complexity science. The four themes identified as barriers to improving knowledge mobilization of IYS were: (traditional scientific practices (incentives for journal publication); organizational obstacles (siloed language and knowledge), change aversion orgs not provided support for change management, and pre-established stakeholder hierarchies (staff not included in change decisions)
Evidence-based Treatment Dissemination Center (EBTDC)	Hoagwood (2014)	Multiple system strategies for scaling EBPs	NY State, USA	A group of policymakers, researchers and consumers have continuously worked together to seek funding, implementation and research opportunities (researcher-practice partnership)	The partnership began with NIMH funding matched by the NYS Office of MH (OMH). The paper isn't entirely clear about when pilots were funded through state dollars or federal research dollars. Most all training efforts were supported through a state training center of excellence (IDEAS center that appears to house the Clinic Technical Assistance Center) that received state funding	Developed a vertical and horizontal network of state policymakers, agency directors, mid-management supervisors, clinicians, family partners, and researchers. Most all strategies included training and consultation. Created EBTDC to train front-line clinicians and supervisors working within clinics, residential treatment centers, and inpatient hospitals on specific EBTs for youth. Trainings and support offered on the following topics: Business practices, EBT trainings, use of EHR for improving clinical practices, parent activation, and hiring of peer specialists	ND	The authors highlight the importance of "liquid networks"—loosely formed multidisciplinary groups that work on the edges of new ideas
PEPP-Montreal	MacDonald (2018)	Protocol, referrals for FEP	Montreal, Canada	ND	This work was supported by multiple grants (CIHR Foundation and NIMH), though limited details provided. Services were paid through public services and free for clients	The article notes the services that were provided but not how those skills were developed. Article mentions a single intake clinician has continuously been used for PEPP, who was internally trained. Intake clinician trainees shadow trained intake clinicians and clinical and introduced to family support group coordinators and other key service partners, though specific training details not included	ND	The article does not address scalability directly but notes that providing simple and quick treatment engagement facilitates service engagement, and facilitates getting to population reach
ACCESS-OM	Malla (2019)	MH system transformation model (includes Early identification, rapid access, continuity of care)	14 diverse sites in Canada	Project's governance structure was designed to meaningfully engage all relevant stakeholder groups in overseeing, administering, and evaluating the transformation of youth mental health care. Activities of the network are managed through an Executive Committee with representation from all sites, two representatives from the national Youth Council and one from the Family and Careers' Council	Federal research and private, blended by intermediary. Funding from Canadian Institutes for Health Research and the Graham Boeckh Foundation. Three years of full funding and two years of reduced "sustainability" funding. Funding covers research staff, one or more clinicians, capital expenses, youth/family engagement in planning, misc. stipends, and IT infrastructure. Funding averages between 290-320 K Canadian dollars per year	The central team assisted each site to identify targets for reduction of unmet needs. Activities at each site vary by local geographic, population size and cultural context although some general principles apply to all sites. ACCESS OM is a strategy for building capacity at the service (site) level and increasing competencies of frontline clinical staff. All clinicians in the integrated service team and evaluation staff at each site receive face to face training focused around the values, ethos and principles of ACCESS OM. This particular training focuses initially on teaching basic mental health assessment skills using the WHO Mental Health Action program (mh-GAP), modified to suit local cultural context. Case vignettes covering a variation of severity of mental health problems are used to provide training in clinical and research evaluations. All aspects of training are provided initially in person with a semi-standard format at each site supplemented by booster sessions through subsequent face to face sessions and webinars	ND	The authors note the importance of early engagement with stakeholders
North Carolina TF-CBT Learning Collaborative	Amaya-Jackson et al.(2018)	TF-CBT	North Carolina, USA	The article mentions that the funders wanted the initiative to focus on underserved areas	Two philanthropy organizations and the state mental health division. The article doesn't note whether funding was used for sites but it appeared to fund all of the preparation and implementation support activities, including organizational preparation, clinician training, and consultation	Clinics were connected to each other through a learning collaborative and used time on calls to brainstorm overcoming barriers to implementation. Training involved three face-to-face sessions (9 mos.), consultation (3 mos.), 1:1 coaching online peer forum/document sharing, and PDSA cycles to support CQI, and a 'senior leader' track to support change management	The project sent out a call for participation to clinicians. Note—many of the clinicians were solo practices	The authors conclude that expert consolation was important for successful implementation (relation between fidelity and decreased PTSD). But note the conflict of interest with coauthors who benefit from delivering TF-CBT in traditional training/consultation formats. The paper also notes that the initiative had a waitlist, suggesting it was not designed for true scale
Continuing Professional Development program	Kiima (2010)	Continuing professional development program to support front line primary care staff to assess, diagnose and manage mental disorders	Kenya	Expanded the Directorate of Mental Health, constructed guidelines for addressing mental health policy within the health service, trainings for district health coordinators on mental health services, establishment of systems for continuing professional development of primary care providers to address mental health. Mental health legislation to reactivate a National Board Kenya Board of Mental Health	Various sources blended by intermediary (WHO). Limited term funding from the UK Dept of International Development, and Nuffeld. The WHO Collaborating Centre, Institute of Psychiatry was the intermediary	The project focused on building mental health access largely through a focus on training nonspecialists on mental health needs and noting that specialist mental health professionals would also need to take on future training of nonspecialists to address mental health needs in non-mental health care settings (primary care, prisons etc.). A leadership component was added to mental health specialist training at existing training programs	ND	The authors note the value of working "within the system" as many donor projects collapse when funding is discontinued. Because the project worked closely with the Ministry of health and other relevant ministries, the system is expected to continue regardless of funding or personalities. The authors also note the importance of coordinating policy activities across multiple sectors and levels, intensive policy dialogue, and evaluation, and working with existing/regular ministry budgets. Further, having a long period of collaboration (10 years) facilitated more sustainable impacts
NY State TA center	Acri (2019)	Technical assistance center for MH professional training and consultation	NY State, USA	The technical assistance centers were the result of the health service system transition to Medicaid Managed Care in 2011 that necessitated changes in fiscal and clinical practices across the behavioral healthcare system	Not clearly described, it appears that the TA centers were funded by the NYS DOH	TA centers provided trainings on a number of topics including both clinical practices and business (evidence-based treatments, clinic practices, business practices, or a mixture of topics. Events were conducted onsite in face-to-face learning communities, off-site via webinars, or a combination of both	Paper briefly mentions "extensive outreach" was done by the TA center; however, this was not described	TA centers can reach across a system of care, such as the public behavioral healthcare service system, and facilitate dissemination of effective practices. The most popular trainings were delivered as webinars (focused on both business and clinical practices)
Rolling Cohort—MTFC	Chamberlain (2012)	MTFC	England	A number of initiatives were enacted to support childhood health and development leading to progressive policy development that laid the ground for a number of different government initiatives (including on one MTFC)	Local children’s services departments responsible for the placement of children in the care system teamed up with their health and education services partners to bid for start-up funding (pump priming grants) to establish MTFC in their local area (set up costs only). National Implementation Team was supported by the government. Government extended funding for three additional years to expand	National Implementation Team worked closely with the MTFC program developers, organized training for staff and foster parents, provided weekly consultation and supervision, attended steering groups with senior managers, organized update events, and received regular audit data and feedback from each site on their development, implementation progress and concerns	ND	Credible local organizations played a critical role in initiating and supporting the scale-up efforts. Highly informed community experts were needed to perform critical functions in the scale-up process
Cascading model—KEEP	Chamberlain (2012)	KEEP	San Diego County, USA	Aims were jointly determined by the HHSA system leaders and the researchers	Grant, a RCT	Train the trainer model (with cohorts)	ND	Credible local organizations played a critical role in initiating and supporting the scale-up efforts. Highly informed community experts were needed to perform critical functions in the scale-up process
School Mental Health Service (SMHS)	Alonge (2020)	SMHP provided by non-specialists	Egypt, Pakistan, Iran	A core team of national champions is identified that facilitate planning, engagement, and advocacy with relevant stakeholders. This national team targets key ministries including education and health, and relevant NGOs at the outset and over the course of implementation	Article mentions external funds for implementation team. Though not clearly described in the article	SMHP implemented through cascade trainings where master trainers (child and adolescent mental health specialists) train national/district trainers, who themselves cascade this training to nominated school staff; with ongoing supervision of non-specialists provided by national/district trainers throughout the process of SMHP training and delivery	Identified national champions, formulated dedicated cross-sectoral implementation teams (including the health and education sector)	Identifying national champions, formulating dedicated cross-sectoral (including the health and education sector) implementation teams, sustained policy advocacy and stakeholder engagement across multiple levels, and effective coordination among education and health systems especially at the local level are among the critical factors for large-scale implementation
Access to Allied Psychological Services (ATAPS)	Bassilios (2014)	ATAPS, a national primary mental health care program	Australia	Government funded program created by the "Better Access" initiative in 2006. Fee-for service rebates for Australia's publicly funded universal health care system was capped and model was revised to allow for more flexible services to at risk populations (including kids with MH disorders)	ND	ND	ND	Example of a large scale system change initiative effectively implemented at the national level to increase access to children's mental health services. Authors note that the ATAPS model, which is predominantly reliant on a GP referral, may not suit all young consumers given that access to services is reliant on their having a GP. This system works well in Australia, with a national health system, but may not in other countries
Skills for Life (SFL)	Guzman (2015)	School-based mental health program	Chile	Established National Program is funded by national department of education in Chile (because this is an established program 15 + years in, they did not describe up strategies (may be in other publications)	Appears to be sustained national program	Article states that Further details about the intervention are provided in a series of manuals (available in Spanish) that are used to train the psychologists who run the workshops	All public and subsidized private primary schools can apply to participate in the program. How they engage and recruit schools was not described	Example of a large-scale mental health intervention successfully implemented in schools and found to be associated with improved behavioral and academic outcomes
SafeCare®	Hurlburt (2014)	SafeCare® an evidence-based child neglect prevention program	Large CA County, USA (not named)	Interest from stakeholders in the child welfare system and the foundation chapter in supporting a system-wide improvement in the area of child maltreatment led to the creation of a “Council” focused on a possible quality/capacity enhancement effort that eventually centered on SafeCare. Council included representatives not simply from county child welfare services and the local foundation, but from community-based non-profit organizations, advocacy organizations, a children’s hospital, EBP developers, and researchers	Not well described. A local foundation supported initial training and development of the seed team. Likely that DHHS also provided implementation funds, but not described	The seed team assumed responsibility for ongoing training of new teams of practitioners that consisted of employees from several local non-profit organizations (called the ICTs). These ICTs form for the express purpose of learning and mastering delivery of the EBP to be implemented, under the guidance of the original seed team	Not discussed, presumably sites were recruited from an initial call for interested providers but the process isn't elaborated on	Six notable issues relating to implementation process emerged from participant interviews, including: (a) initial commitment and collaboration among stakeholders, (b) leadership, (c) communication, (d) practice fit with local context, (e) ongoing negotiation and problem solving, and (f) early successes. The authors note the value of a phased roll out to incrementally gain buy in; and then using a "seed" team for supporting implementation allowed for adaptation and problem-solving, championing
Multi County MTFC Implementation (CAL-40 study)	Palinkas et al.(2013), Saldana & Chamberlain (2012)	Multidimensional Treatment Foster Care (MTFC)	CA and OH states, USA	ND	Large randomized implementation trial, appears to be through NIMH, NIH funds	The community development team approach uses a combination of peer-learning networks and individual coaching to build the capacities of counties to offer MTFC. The implementation support strategies are multifaceted: need/benefit analysis, training, coaching, peer coaching. evaluation	ND	The peer-to-peer coaching model was critical for supporting adaptations to the model that, had the developers been the only TA provider, would have stalled/inhibited implementation. The approach put implementation specialists and developers on equal footing to support tailored approaches to implementing the intervention
Family Check-up (FCU)	Mauricio et al. (2021))	FCU, a brief, assessment-driven intervention	Gothenburg Sweden	Local Initiative. Precipitated by initiative prepared for National Board of Health & Welfare. Established center to develop plan for implementation and roll out	Not clear where funding came from. Appears to be locally supported but not described	The Center collaborated with the FCU developer to train and certify Center staff responsible for disseminating the FCU and providers in agencies implementing the program. Program developer visited twice (2 years apart) to conduct training workshops. Process supported a hybrid bottom-up, top-down adaptation that balanced the Swedish purveyor’s autonomy and cultural expertise with guidance from the United States	Collaboration involved in-person and virtual meetings to iteratively adapt training. Collaboration also involved a bi-annual international conference to unite and support teams in different countries	Implementation frameworks can be used to guide cross-country transport of interventions by helping communities build implementation readiness. Promoting a culture of prevention depends on a community’s readiness to use evidence-based practices to address problems preventively rather than reactively
Empowering Parents Empowering Communities (EPEC)	Day (2022)	EPEC is a task sharing, peer-led, group based parenting approach	England	Not described	Funded by the UK Early Years Social Action Fund; NESTA; and Department for Culture Media & Sport South London; and the Maudsley NHS Foundation Trust	Three inter-related phases: Phase 1: Hub engagement and initial set up Phase 2: Hub staff training in Being a Parent manualized content and methods, recruitment and training, supervision and quality assurance, and engagement of local stakeholders and communities Phase 3: Hub implementation. Each hub established pathways to engage local parents. National consultants used manualized quality standards to appraise hub implementation, problem-solve, and support site scaling using ongoing digital and face-to-face contact and quarterly collaborative Hub learning and exchange events	Hub host organizations were selected because of compatibility between their local strategic priorities, operational resources, parenting and peer expertise, and population needs, and EPEC aims and programme theory	Benchmarking comparisons provided an efficient, pragmatic, and low-cost method to compare scaling program with trial performance
Youth FORWARD	Bond (2022)	Youth FORWARD designed to provide EBP mental health services while advancing goals shared with government and development actors	Sierra Leon, 12 chiefdoms	Details are not well described "Youth FORWARD includes partnerships across a range of stakeholders in policy and investment, service delivery, research, and capacity building"	YRI was originally developed to be delivered in a different setting. Sierra Leone's Economic Development plan, partnered with World Bank, included policy actions to support youth entrepreneurship and skills development. YRI was incorporated with a job/skills training program for youth	Used a Collaborative Team Approach (CTA). The seed team consisted of three YRI experts who provided ongoing training, coaching and support to YRI facilitators as the intervention moved from pilot to scale-out. Fidelity to evidence-based practices was achieved by meeting individually with YRI facilitators before each YRI session to ensure they were prepared and through weekly supervision meetings with YRI facilitators	Not described in this paper	Research and interventions should be contextualized with careful consideration of risk and protective factors across all levels of the social ecology. Psychosocial interventions should be based on locally identified needs, rather than externally imposed services or assumptions, identifying priorities through community-based approaches and collaboration with local service providers and community advisory boards. Educational and employment programs should be considered and explored as alternative delivery platforms to effectively deliver mental health services by community based lay workers, addressing the limited human resource and capacity challenges in post-conflict settings

Scaling approach used by each initiative included in the review and reported scaling outcomes. Scale “up” = intentional efforts to influence policy, Scale “out” = resources devoted to communicating and supporting buy in, Scale “in” = site capacity building, Scale “down” = efforts to devolve power to local communities, ND = no data available

**Table 4:** Scale up and implementation strategies

Program	Scalable unit	Reach aim	Achieved reach	Framework	Evidence-informed	Designed to local needs	Endorsement from policy/system leaders	Clinical supervision	Internal adaptation teams	Organizational peer support	Leadership support	Feedback and evaluation	Stakeholder engagement	Policy guidelines	Self-guided resources	Train the trainer	Expert consultation
On Track New York	Coordinated Specialty Care for Early Episode Psychosis	Entire state	21 sites/unknown, estimated 2000 clients left unserved	ND	Yes	ND									X		X
LA County Mental Health Project	Treatment Protocols	Entire county	120 agencies/120	References EPIS but unclear application. Implementation strategy seemed rushed due to forced funding shortfall	Yes	ND	X	X				X				X	X
BASIC—Building and Sustaining Interventions for Children	Group based TFCBT adapted as a task shifting model (delivered by both education and health sectors workers	Primary schools in 10 villages, Western Kenya	10 villages (first cohort of RCT to train 240 counselors from 40 communities	ND	Yes	Yes	X	X	X							X	X
Multiple Family Group for Strengthening Families	The 4 Rs and 2 Ss Multiple Family Group for Strengthening Families	Large USA City	Yes, but working with an existing system structure	Blended multilevel implementation strategy	Yes	No	X	X	X								X
CBITS Connecticut middle schools	CBITS	State Middle school system	350 kids received CBITS during a 2 year period. 73 CBITS group conducted in total	Implementation guided by Proctor and Colleagues (2013). Learning collaborative model, engaged leadership in introductory meetings. Used measurement feedback, QA system	Yes	Yes					X						X
FRAYME Network	Network designed to accelerate the adoption and scaling up of IYS	Entire country	ND	Describes an integrated knowledge mobilization strategy	Yes	Yes											
Evidence-based Treatment Dissemination Center (EBTDC)	Multiple system strategies for scaling EBPs	Entire State	ND	Not formally, the paper predated the explosion of implementation science frameworks, but the authors reference diffusion of innovations as general support for their approaches	Yes	Yes											X
PEPP-Montreal	Protocol, referrals for FEP	Entire City	Yes?, 1750 referrals over 13 years (no comparison)	ND	ND	Yes							X				
ACCESS-OM	MH system transformation model (includes Early identification, rapid access, continuity of care)	Country	ND	The paper doesn't not whether ACCESS-OM's strategy for replication is informed by an explicit implementation strategy apart from building in research and evaluation capacity (which is likely providing "soft" implementation guidance via developmental/utilization focused evaluation)	Yes	Yes			X		X	X	X				
North Carolina TF-CBT Learning Collaborative	TF-CBT	State system	ND	The implementation plan was very robust, pulling from EPIS and other implementation frameworks and included multiple layers of support (leadership, clinician training, peer learning, PDSA cycles, performance metrics)	Yes	No									X		X
Continuing Professional Development program	Continuing professional development program to support front line primary care staff to assess, diagnose and manage mental disorders	Entire country	ND	The process began with a Situational Appraisal aligned with the model developed by Murry and Fren. The process also included a sustained policy dialogue on mental health with multiple departments in Kenya. This resulted in Mental Health Policy Guidelines. Mental health was then integrated into health sector reform plans, Health Interventions, national essential drugs list, and the health management information system	ND	Possibly					X		X	X		X	
NY State TA center	Technical assistance center for MH professional training and consultation	State system	Focused on training only, but did increase reach for workforce. Between 2011 and 2015, 460 (92.6%) of all New York State mental health clinics attended a training	Not clear, though the article cites multiple articles focused on implementation science	Yes	ND					X						
Rolling Cohort—MTFC	MTFC	Entire country	ND	Rolling Cohort Model, did not adhere to any particular theoretical framework	Yes	Yes	X				X	X	X				X
Cascading model—KEEP	KEEP	Country	ND	Cascading Model, did not adhere to any particular theoretical framework	Yes	Yes					X	X				X	X
School Mental Health Service (SMHS)	SMHP provided by non specialists	Multiple countries	ND	They used CFIR framework (or at least certain concepts from CFIR). Also used a theory-based framework (Theory of change; ToC) methodology was used to identify pathways and strategies for implementation at scale. Adapted approach previously applied as a tool to guide evaluation of complex mental health interventions (De Silva et al., 2014; Asher et al., 2015; Chibanda et al., 2016; Breuer et al., 2018)	Yes	Yes	X									X	
Access to Allied Psychological Services (ATAPS)	ATAPS a national primary mental health care program	Entire country	Yes, served a large number of kids during a 10 year period (51,716 clients, 245,707 total sessions)	ND	Yes	ND	X										
Skills for Life (SFL)	School-based mental health program	Entire country	Yes?, 1/5 of schools opted to participate (1637 schools). 1007 students referred to specialists (784 students engaged in treatment). 1/2 of those students also attended workshops	Not discussed. Note—the "Skills for Life" program was based on the 3-tiered model recommended by the WHO (not clear if this includes implementation or scalability recommendations)	Yes	Yes	X										X
SafeCare®	SafeCare® an evidence-based child neglect prevention program	Entire country	ND	Described application of the Interagency Collaboration Team (ICT) process model, which builds on EPIS	Yes	Yes				X						X	
Multi County MTFC Implementation (CAL40 study)				Used the Community Development team (CDT) approach to scale up the EBP. The CDT approach involves peer-to-peer interactions among counties who are undergoing the training in the steps required to implement this complex program	Yes	ND				X	X	X	X				
Family Check-up (FCU)	FCU, a brief, assessment-driven intervention	Entire City	ND	EPIS and the determinants-based implementation drivers framework (NIRN)	Yes	Yes	X	X		X		X					X
Empowering Parents Empowering Communities (EPEC)	EPEC is a task sharing, peer-led, group based parenting approach	Multiple sites within Country	Six hundred and eighty-four parents (73.5%) completed the course across the 15 sites	ND—this appears to be part of the plan. But no specific strategy mentioned in the paper	Yes	Yes						X					X
Youth FORWARD	Designed to provide EBP mental health services in combination with a career development program	Multiple sites within Country	Pilot and scale up programs reached 1783 participants	EPIS	Yes	Yes		X	X			X				X	X

Implementation strategies used and achieved reach for each initiative included in the review

*ND* no data available

## Results

The studies identified for the review were primarily conducted in the United States (14 papers, 10 unique initiatives), followed by Canada (3 papers/initiatives), the United Kingdom (2 paper/initiative), Kenya (2 papers/initiative), Siera Leon (1 paper/initiative), Sweden (1 paper/initiative), Chile (1 paper/initiative), Australia (1 paper/initiative), and a paper describing efforts in Egypt, Pakistan and Iran (1 paper/initiative).

Studies focused on either improving access to mental health services (11 papers), or improving the quality of existing services (14 papers), reflecting both scale-up (new services or expanded reach) and spread (improving quality in an existing system). Among studies focused on improving access, six of the studies focused on improved referral and coordination of mental health services; and five studies focused on the use of nontraditional mental health providers. All of the studies focused on intervention spread (innovation diffusion) within traditional healthcare environments were conducted in the United States. All of the non-U.S. country studies focused on improved service referral/coordination or the use of nontraditional service providers (e.g., lay facilitators, school personnel) rather than improving quality of care within the existing healthcare system.

### Theoretical Models and Frameworks

Only one study explicitly referenced a model for scale-out, the Collaborative Team Approach (CTA; Bond et al., 2022), while another paper referenced a framework designed to achieve population reach (RE-AIM Gaglio et al., 2013; Mascayano et al., 2019)). About half of the papers (11) cited an implementation framework as a core design consideration or as a reference-point for clinical training and organizational support efforts. The most cited framework was Exploratory, Planning, Implementation, Sustainment (EPIS, 6 efforts) (Moullin et al., 2019), followed by the Interactive Systems Framework (Wandersman et al., 2008) (1 effort), the Consolidated Framework for Implementation Research (Kirk et al., 2016) (1 effort), Theory of Change (TOC, 1 effort), National Child Traumatic Stress Network Learning Community (Pynoos et al., 2008) (1 effort), and Diffusion of Innovations (1 effort) (Greenhalgh et al., 2004).

### Type of Study and Outcomes

None of the studies involved a controlled evaluation of scale-up strategies. One study included the use of a quasi-experimental design with embedded benchmarking to evaluate the scaling-up and scaling-out of a program (Day et al., 2022). Nineteen of the 22 studied initiatives included descriptive analyses, pre/post designs with no comparison, or case studies. Five initiatives reported within client (pre/post) clinical improvement during the period of scale-up: (Mascayano et al., 2019; Southam-Gerow et al., 2013; Hoover, et al., 2018; Amaya-Jackson et al., 2018; Guzmán et al., 2015). Nine studies reported on reach (regional population coverage; either workforce population or client population): Bond et al., 2022; Day et al., 2022; Hoover et al., 2018; Mascayano et al., 2019; MacDonald et al., 2018; Acri et al., 2019; Alonge et al., 2020; Bassilios et al., 2014; Guzmán et al., 2015).

### Scope of Implementation

The smallest reach included 10 villages in Eastern Kenya and the broadest intended reach was multi-national among three countries (Egypt, Pakistan, Iran). The scope of implementation included studies within a single city (4), single county or district (4), single state (5), multiple counties, states, provinces, regions (5), and national/country level (4).

### Policy Strategies (Scaling “Up”).

Policy strategies (scaling “up”) described how the implementation effort intentionally engaged policy and system norms to encourage sustainability. The approaches yielded three major themes: First, the largest-scale efforts (as defined by target population) were facilitated by third party intermediaries (non-governmental) that had pre-existing relationships with government entities or, in one case, brokered the partnerships needed to achieve significant system changes. Second, securing financing from routine payment mechanisms was a key driver of sustainability (not just single investments for start-up). Third, large-scale implementation was associated with multi-sector planning efforts involving vertical (policy, provider, consumer) and horizontal (multiple departments and sites) representation.

*Third party intermediaries*. Eleven of the studies included a pre-established or concurrently funded intermediary that supported policy efforts and/or implementation. Six of the studies described efforts in which a third-party intermediary (e.g., university, nonprofit) worked with policymakers prior to identifying and implementing the initiative described in the paper. This suggests long-term partnerships are conducive to large-scale change, although sustained changed could be not inferred from the available literature. For example, Malla et al. (2019) describes a national effort to transform youth serving systems, ACCESS-OM, facilitated by a central organizing intermediary to support local system change effort, funded with philanthropic and government funds, that engaged local policymakers prior to implementation.

*Routine financing.* Identifying opportunities for routine, sustainable financing of new services was noted as a policy strategy for supporting implementation in four studies. The Los Angeles County mental health expansion of evidence-based practices (the subject of four papers in the review, Rodriguez et al. (2018); Reding (2018); Brookman-Frazee et al. (2016); Southam-Gerow et al. (2013)) was facilitated by state funding requiring the delivery of “innovative” mental health programs. Bassilios et al. (2014) describes the Australian government’s establishment of billing codes for supportive, nonpsychotherapy services as a funding incentive to implement and sustain integrated care services.

*Multi-sector planning*. The successful engagement of multiple sectors in planning (number of sectors and partners) was a repeatedly noted factor in achieving reach in scaling efforts well as local implementation success. For example, Halsall et al. (2020) describes the importance of multi-sector planning (government, healthcare system, youth/family, philanthropy) as a start-up strategy when preparing new project implementation sites for FRAYME, a youth oriented, mental health system reform effort focused on increasing access to care. Hoagwood et al. (2014) similarly describes the value of multi-sector planning (state government, providers, researchers, youth/families) in maintaining the productivity of a university-government collaboration focused on improving the quality of children’s mental health care in New York State. Day et al. (2022) describes how the Youth Readiness Intervention (YRI) program was funded by combined with another job/skills training program to form Youth FORWARD because Sierra Leone’s Economic Development plan included policy actions to support youth entrepreneurship and skills development.

### Devolving Control to Local Entities (Scaling “Down”) Strategies

Devolving control to local entities (scaling “down”) was only broadly defined in the Sanchez Rodriguez framework. Consequently, in coding for this category, we looked for instances of “hand off” between the originating consultants to local community decision-making. This excluded instances of train-the-trainer models in which trainers were not given permission or guidance to independently modify or adapt models or intervention to suit local circumstances. We observed efforts to encourage local control occurring most clearly in descriptions of the FRAYME, ACCESS-OM, FCU, EPEC, and Youth FORWARD efforts, all of which are approaches aiming to substantially restructure CYMHS services, increase population access, prioritize receiving care in nontraditional environments when possible, and deliver ‘youth-friendly’ services. In these models, communities are provided with principles of care and service delivery and are then supported with technical assistance and funding for onsite planning and evaluation to develop local system approaches. Three additional studies described strategies that also appeared to fit within the definition of scaling “down.” The SafeCare implementation described by Hurlburt et al. (2014) used seed teams train by the expert clinical team to then train new, incoming teams using problem-solving frameworks to generate novel approaches to local implementation. The Multiple Family Group for Strengthening Families initiative described by Choy-Brown et al. (2020) supported “internal adaptation teams” to support site-specific localization. The roll out of the MultiDimensional Treatment Foster Care in multiple counties used peer coaching and tools for analysis and evaluation to support initial planning for implementation and ongoing quality support. The Youth Readiness Intervention described by Bond et al. (2022) used the Collaborative Team Approach (CTA) as a scale-out strategy to pilot and scale-out the program and to shift decision making and ownership to community stakeholders (Bond et al., 2022).

### Strategies Used for Capacity Building (Scaling “In”)

Capacity-building funding to support initial implementation efforts (scaling “in”) was diverse, coming from federal service contracts, federal research grants, state or county service contracts, and philanthropy. Ten of the papers reported blended funding and the most common approach included a blend of philanthropy and government funds (8 approaches), pointing towards a key role for philanthropy in supporting system and policy-level innovation. Longer term initiatives were either fully funded by government or were facilitated by intermediaries that blended funding sources over time and acted as a convening partner for policy and funding stakeholders.

*Specific capacity building strategies.* Papers varied considerably in the degree of detail they provided about engagement, training and support strategies. We drew from additional papers and online searches describing initiatives to supplement the information found in the included papers. Even so, we presume our review is not a comprehensive review of strategies used in these efforts. As a result, we present this summary as preliminary information about the kinds of strategies that appear to be common across scale up in CMHYS efforts rather than definitive of all strategies used in the reviewed initiatives. A total of twelve scaling “in” (site capacity building) implementation strategies were identified across the 22 initiatives (Table 4): Design to fit local needs (14 papers), Endorsement from policymakers (8), Clinical supervision (5), Internal adaptation teams (3), Organizational peer support (3), Leader support (6), Feedback and evaluation (8), System partner engagement (5), Policy guidelines (1), Self-guided resources (1), Train the trainer (7), Expert delivered consultation (11).

*Localization strategies.* Efforts to design around local needs tended to focus on system-level adaptations (e.g., engaging local partners to plan for implementation) rather than adaptation of curricula. The level of shared planning between research and system partners varied as did the boundaries and available options for decision-making. For example, the North Carolina TF-CBT project engaged system partners in selecting TF-CBT training from a list of options, after which the research partners facilitated an implementation strategy that preserved the original curricula in sites of practice. Whereas, mental health policy guidelines in Kenya designed to direct local authorities on how to train frontline providers were developed de novo rather than selected from a list through an iterative process beginning with a Situational Appraisal approach (Kiima & Jenkins, 2010).

### Strategies for Engaging Multiple Sites (Scaling “Out”).

Descriptions of how efforts engaged multiple sites to achieve reach (scaling “out”) were the thinnest compared to the other three scalability categories. Seven initiatives provided enough information to warrant coding. Of these initiatives, strategies sorted into three categories: (1) Financial; (2) Soft pressure, and; (3) Marketing. Two initiatives used financial levers to engage providers. The Los Angeles County Mental Health Project capitalized on a temporary requirement that state funds be used to support “innovative” mental health services to require all existing public mental health organizations to select a minimum of one evidence-based training from a pre-approved list (Brookman-Frazee et al., 2016). The Rolling Cohort MTFC initiative released RFPs for developmental funds on top of reimbursement to incentivize implementation (Chamberlain et al., 2012). Two initiatives described “soft pressure” methods. The BASIC study in Kenya recruited villagers familiar with the intervention to speak about the project in other villages (Dorsey et al., 2019). The School Mental Health Service (SMHS) in Egypt, Pakistan, Iran identified national champions and cross-sector teams that identified sites and championed implementation (Alonge et al., 2020). Three initiatives described marketing efforts in which invitations to participate in free training were widely distributed and relied on organizations to contact the lead organization to obtain training (Choy-Brown et al., 2020; Amaya-Jackson et al., 2018; Hoagwood et al., 2014).

## Discussion

We conducted this scoping review to examine research approaches and trends in the CYMHS scale up literature. We had three primary aims: (1) To understand the frameworks and models guiding real world efforts; (2) To examine preliminary clinical and scale-up outcomes; (3) To document scale up strategies with an eye to capturing unique features observed in scaling up child and youth-focused services. Regarding the first question, we observed that real-world efforts in CYMHS scale up are not robustly informed by scalability frameworks. The methods and outcomes used in the included studies were highly variable and precluded our ability to draw conclusions about the comparative effectiveness of scale-up approaches. Very few of the studies collected outcomes most relevant to scale-up (access, coverage, changes in clinical competency). Scale-up successes noted the need to adopt culturally responsive implementation methods reflecting periods of reflection, deliberation and adaptation. We expand on these findings, below, and offer recommendations for the future study of scale up efforts in child and youth mental health services.

Existing scale-up frameworks differ in their details but are consistent in recommending methods that tailor the unit of scaling (e.g., a program or protocol) to the local system and/or client population. Less than half of the initiatives (10) in our review selected or developed clinical training protocols to fit the needs of the specific region identified for scaling. Among these studies, the descriptions of clinical training were thin and did not appear to involve the phases of testing advised by scaling frameworks. A few studies noted that practices were selected for certain elements related to fit. For example, Choy-Brown et al. (2020) notes that the MSFS program was selected because it was Medicaid reimbursable. No study reported using a systematic assessment of different programs based on a formal review of scalability factors.

A recent systematic review of scalability assessments (Ben Charif et al., 2022) notes that without consideration of these local factors and the end-users/community in determining fit, scale-up efforts can have unintentional negative impacts. Several assessments assessing the potential scalability of interventions are available. The Intervention Scalability Assessment Tool (ISAT, Milat et al., 2020) can be used to guide implementation efforts responsive to conditions of the local context. An increasing number of CMHYS clinical tools are being developed and tested for the express purpose of scalability, including task-shifting (Dorsey et al., 2019), caregiver-training protocols (Heyne et al., 2002), low-burden clinician training approaches (Tchernegovski et al., 2015), and digital health resources (Hollis et al., 2017; Moltrecht et al., 2022). The observed lack of use of scalability frameworks and assessments in the current review suggests the CYMHS field may be lagging behind other health fields in integrating the scalability as an implementation factor when innovating and testing system-level quality improvements efforts. The dominant approach observed in this review was to begin with the clinical intervention and then attempt to take the approach to scale in a willing community. This approach often favors early adopters and implementation sites with higher resources and can underserve communities and systems where there is the greatest need and potential for impact (Sánchez-Rodríguez, 2019).

Findings suggests adjusting macro factors (funding and policy) to reflect the specific clinical needs of child and youth populations. For example, the MDTFC roll out in the UK used a rolling RFP with start-up funds to incentivize local sites to implement the program, knowing that the full costs of delivering the family-intervention (requiring home visits and other coordination not typical for traditional outpatient care) would devolve to the local sites over time. While only a few studies included information on scaling funding strategies specific to CYMHS, studies in our review that achieved significant policy and system integration, overall, were supported by long-term partnerships (e.g., Alonge et al., 2020; Halsall et al., 2020; Hoagwood et al., 2014; Kiima & Jenkins, 2010) involving multiple sectors (funders, government, research, practice). The efforts aiming at significant scale tended to blend private (e.g., humanitarian, or philanthropic) and government funds (e.g., state, or country). While the studies we reviewed did not provide deep descriptions of the individual capacities or strategies that made long-term partnerships possible, the broader literature on knowledge brokering and research-practice partnership suggests productive university or research-policy partnerships require professionals who have specialized collaborative skill sets, developed over years of partnership with system and policy partners (Jessani et al., 2016; Welsh, 2021). Some of these noted skills include the ability to communicate clearly across audiences (Bennett & Jessani, 2011), the ability to synthesize complex information from multiple sources (Lomas, 2007; Welsh, 2021), having a policy focus and political savvy (Jessani et al., 2016), and effective leadership skills (Jessani et al., 2016).

Finally, measures of scale-up were largely missing in the studies we reviewed. Future study of scale-up in CMHYS will benefit from the standardization of measurement constructs. Wide variability in methods, measures and analytic approach in this review precluded us from drawing conclusions about the relative benefit of specific scale-up strategies. One study found the use of benchmarking, a quasi-experimental design using a comparison group derived from previously published RCT results, as a useful and feasible strategy for real world scale up initiatives (Day et al., 2022). We recommend that scale up studies clearly report the scope of the target population and the achieved reach in client contact and organizational partners, following, for example standards in the RE-AIM framework (Glasgow et al., 1999). We additionally recommend that researchers studying methods to increase clinical skills among clinicians use and report measures of clinical competence (Cooper et al., 2015) in order to facilitate comparisons across different training approaches.

### Strengths and Limitations

To our knowledge, this is the first review of scale up for child and youth mental health services with a specific focus on implementation strategies. As such, it provides a unique contribution to a health area of contemporary and urgent interest. We note a few limitations as well. This study is limited to articles that used eligible terms for scale up in titles and abstracts. Consequently, this analysis may be missing articles not framed in terms of scale up but nonetheless including descriptions of strategies or outcomes of interest to this paper. The conclusions we draw are, therefore, limited to a narrow literature of studies explicitly focused on taking quality CYMHS to scale. We also attempted to draw from the gray literature and found similar issues of fit with the scope of this paper. In particular, the gray literature on CYMHS is dominated by policy and clinical guidelines for scaling services rather than detailed descriptions of efforts and outcomes we could use to inform our primary aims. However, we recognize that this review underrepresents the scope of actual real-world efforts to take CYMHS to scale.

### Conclusions

This study of scale-up efforts in child and youth mental health services revealed a number of innovative and diverse efforts occurring around the globe. While the diversity of efforts made direct comparisons infeasible, we observed commonly stated values and approaches. In particular, we noted a high frequency of efforts to tailor and adapt strategies across sites as well as “hand off” local control of implementation over time. The endorsement and visible championing by policy makers was also common and noted by authors as a critical feature of scale-up. The field will need to apply more standard measures and frameworks to advance collective knowledge of CYMHS scale-up over time.

## Appendix A: Search 1 terms

### Example search string in PubMed

(("Child" OR "Adolescent" OR "children’s" OR "children" OR "youth" OR "young people") AND ("Mental health" OR "psychological" OR "Psychiatric" OR "Psychiatry" OR "Psychological" OR "neurodevelopmental" OR "neurodevelopment") AND ("scale up" OR "scale-up" OR "scaled up" OR "scaled-up" OR "scaling up" OR "scaling-up" OR "scaling" OR "scalable") AND ("Case study" OR "feasibility" OR "evaluation" OR "good practice" OR "outcomes" OR "trial" OR "review" OR "study") AND ("2000/01/01"[Date—Publication]: "3000"[Date—Publication])).

## Appendix B: Secondary LMIC terms

### Example search string in PubMed

Included in title only:

"Mental health" OR "psychological" OR "Psychiatric" OR "Psychiatry" OR "Psychological" OR “emotional disorder*” OR “emotional problem*” OR “behavioural disorder*” OR “behavioural problem*” OR “behavioral disorder*” OR “behavioral problem*” OR “conduct disorder*” OR “psychosis*” OR “eating disorder*” OR “anorexia” OR “bulimia” OR depressi* OR anxi* OR trauma* OR distrupti* OR psycho*

Included in title/abstract/keywords: "Child" OR "Adolescent" OR "children’s" OR "children" OR "youth" OR "young people" AND "Case study" OR "feasibility" OR "evaluation" OR "good practice" OR "outcomes" OR "trial" OR "review" OR "study" AND "access to care" OR "improve access" OR "one stop shop" OR "early interventions" OR "integrated care" OR "collaborative care" OR "care coordination" OR "multi-sector" OR "multisectoral" OR "community-based" OR "community based" OR "care transformation" OR "system transformation" OR "primary health" OR "task shifting" OR "lay providers" OR "engagement" OR "participation" OR "inclusion" OR "quality care" OR "care standards" OR "quality standards" OR "high-quality" OR "high quality" OR "child-centred" OR "family-centred" OR " adolescent-centred" OR "youth-centred" OR "person-centred" OR "child-centered" OR "family-centered" OR " adolescent-centered" OR "adolescent-responsive" OR "youth-centered" OR "person-centered" OR " holistic" OR "equitable" OR "human rights" OR "recovery" OR "evidence-based" OR "evidence based" OR "building capacity" OR "task-shifting" OR "training hubs" OR "policy development" OR "multi-disciplinary care" OR "expanding mental health" OR "access" OR "low-cost" AND "2000/01/01"[Date - Publication] : "3000"[Date - Publication]

MeSH Terms: AND "LMIC” OR “low-income countries” OR “middle-income countries” OR “middle- income country” OR “low-income country” OR “low- middle-income countries” OR “low-middle income country” OR low resource* OR Afghanistan OR Albania OR Algeria OR American Samoa OR Angola OR Antigua And Barbuda OR Argentina OR Armenia OR Aruba OR Azerbaijan OR Bahrain OR Bangladesh OR Barbados OR Republic Of Belarus OR Belize OR Benin OR Bhutan OR Bolivia OR Bosnia And Herzegovina OR Botswana OR Brazil OR Bulgaria OR Burkina Faso OR Burundi OR Cabo Verde OR Cambodia OR Cameroon OR Central African Republic OR Chad OR Chile OR China OR Colombia OR Comoros OR Democratic Republic Of The Congo OR Congo OR Costa Rica OR Cote D’ivoire OR Croatia OR Cuba OR Cyprus OR Czech Republic OR Djibouti OR Dominica OR Dominican Republic OR Ecuador OR Egypt OR El Salvador OR Equatorial Guinea OR Eritrea OR Estonia OR Swaziland OR Ethiopia OR Fiji OR Gabon OR Gambia OR Georgia (Republic) OR Ghana OR Gibraltar OR Greece OR Grenada OR Guam OR Guatemala OR Guinea OR Guinea Bissau OR Guyana OR Haiti OR Honduras OR Hungary OR India OR Indonesia OR Iran OR Iraq OR Jamaica OR Jordan OR Kazakhstan OR Kenya OR Democratic People’s Republic Of Korea OR Republic Of Korea OR Kosovo OR Kyrgyzstan OR Laos OR Latvia OR Lebanon OR Lesotho OR Liberia OR Libya OR Lithuania OR Macau OR Republic Of North Macedonia OR Madagascar OR Malawi OR Malaysia OR Indian Ocean Islands OR Mali OR Malta OR Micronesia OR Palau OR Mauritania OR Mauritius OR Mexico OR Moldova OR Mongolia OR Montenegro OR Morocco OR Mozambique OR Myanmar OR Namibia OR Nepal OR Netherlands Antilles OR Nicaragua OR Niger OR Nigeria OR Oman OR Pakistan OR Panama OR Papua New Guinea OR Paraguay OR Peru OR Philippines OR Poland OR Portugal OR Puerto Rico OR Romania OR Russia OR Rwanda OR Samoa OR Sao Tome And Principe OR Saudi Arabia OR Senegal OR Serbia OR Seychelles OR Sierra Leone OR Slovakia OR Slovenia OR Melanesia OR Somalia OR South Africa OR South Sudan OR Sri Lanka OR Saint Kitts And Nevis OR Saint Lucia OR Saint Vincent And The Grenadines OR Sudan OR Suriname OR Syria OR Tajikistan OR Tanzania OR Thailand OR Timor Leste OR Togo OR Tonga OR Trinidad and Tobago OR Tunisia OR Turkey OR Turkmenistan OR Uganda OR Ukraine OR Uruguay OR Uzbekistan OR Vanuatu OR Venezuela OR Vietnam OR Middle East OR Yemen OR Yugoslavia OR Zambia OR Zimbabwe OR Africa South Of The Sahara OR Africa, Central OR Africa, Northern OR Africa, Southern OR Africa, Eastern OR Africa, Western OR West Indies OR Indian Ocean Islands OR Caribbean Region OR Central America OR Latin America OR South America OR Asia, Central OR Asia, Northern OR Asia, Southeastern OR Asia, Western OR Europe, Eastern OR Developing Countries
